# The Dual Roles of Nano Zero-Valent Iron and Zinc Oxide in Antibiotics Resistance Genes (ARGs) Spread in Sediment

**DOI:** 10.3390/ijerph19159405

**Published:** 2022-07-31

**Authors:** Ling Luo, Dahang Deng, Xin Zhao, Hairong Hu, Xinyi Li, Jidong Gu, Yan He, Gang Yang, Ouping Deng, Yinlong Xiao

**Affiliations:** 1 College of Environmental Sciences, Sichuan Agricultural University, Chengdu 611130, China; dengdahang@stu.sicau.edu.cn (D.D.); zhaoxin1@stu.sicau.edu.cn (X.Z.); huhairong@stu.sicau.edu.cn (H.H.); li_xinyi@stu.sicau.edu.cn (X.L.); heyan@sicau.edu.cn (Y.H.); yg8813@sicau.edu.cn (G.Y.); 2 Environmental Science and Engineering, Guangdong Technion-Israel Institute of Technology, 241 Daxue Road, Shantou 515063, China; jidong.gu@gtiit.edu.cn; 3 College of Resources, Sichuan Agricultural University, 211 Huimin Road, Chengdu 611130, China; ouping@sicau.edu.cn

**Keywords:** nanoscale zero-valent iron (nZVI), zinc oxide nanoparticles (nZnO), tetracycline resistance genes (tet-ARGs), class 1 integron (*intI1*), tetracycline

## Abstract

Nanoparticles (NPs) are widely used and ubiquitous in the environment, but the consequences of their release into the environment on antibiotics resistance genes (ARGs), microbial abundance, and community, are largely unknown. Therefore, this study examined the effect of nano zero-valent iron (nZVI) and zinc oxide (nZnO) on tetracycline resistance genes (tet-ARGs) and class 1 integron (*intI1*) in sediment under laboratory incubation. The coexistence of NPs and tetracycline (TC) on tet-ARGs/*intI1* was also investigated. It was found that nZVI and nZnO promoted tet-ARGs/*intI1* abundance in sediment without TC but reduced the inducing effect of TC on tet-ARGs/*intI1* in sediment overlaid with TC solution. Without TC, nZVI, *intI1*, and the bacterial community could directly promote tet-ARGs spread in nZVI sediment, while *intI1* and bacterial abundance were the most directly important reasons for tet-ARGs spread in nZnO sediment. With TC, nZVI and bacterial community could reduce tet-ARGs abundance in nZVI sediment, while nZnO and bacterial community could directly promote tet-ARGs in nZnO sediment. Finally, these findings provided valuable information for understanding the role of NPs in promoting and reducing ARGs in the environment.

## 1. Introduction

Antibiotic resistance genes (ARGs) in the environment have recently attracted much attention due to their risk to human health and food security [1,2]. The mortality caused by antibiotic resistance is 700,000 annually and may approach 10 million by 2050 [3]. Thus, ARGs have been proposed as emerging environmental pollutants and have received broad observations on their distribution in various environments, such as soils, sediments, waters, and even the air [4,5,6,7,8,9,10]. Under this circumstance, reducing the abundance of ARGs in the environment is necessary to protect human health.

Reducing ARGs is a direct and effective way to control the risk imposed by ARGs on human health [11,12,13]. Currently, efforts have been mainly focused on reducing ARGs before they enter the environment (i.e., source control). For example, technologies such as composting, alkaline treatment, Fenton treatment, membrane filtration, and others, have been applied to control the abundance of ARGs, and several technologies are efficient in lowering ARGs [14,15,16,17]. However, technologies for reducing ARGs that already existed in the environment (i.e., site remediation) have been less investigated. Notably, once ARGs enter the environment, eliminating them is difficult. For example, if pathogens in the environment acquire these extra ARGs, current antibiotics may be ineffective in controlling and killing pathogens, resulting in unnecessary mortalities [18,19,20]. Therefore, site remediation technologies are necessary to reduce ARG risks in the environment.

In recent decades, nanoparticles (NPs) have been frequently applied to site remediation directly in the environment [21,22]. For example, NPs, such as nano zero-valent iron (nZVI), have been widely applied for remediation of organic and inorganic contaminants in soil and groundwater due to their large surface area and good deliverability [21]. However, on the other hand, NPs are also reported to affect biota due to their potential toxicity [23,24]. For instance, nZVI was reported to diminish soil microbial biomass [25,26], and nano zinc oxide (nZnO) also showed the ability to reduce bacterial abundance in soil [27]. Many NPs exhibit antibacterial effects and are used to combat antibiotic resistance for medical care purposes [28]. From this aspect, applying NPs into the environment may reduce bacterial growth/abundance and thus ARGs through vertical gene transfer (i.e., VGT) [29,30]. Nonetheless, several studies have suggested that NPs (e.g., nZnO, copper NPs, and sphalerite NPs) promoted the dissemination of ARGs across bacterial genera through horizontal gene transfer (i.e., HGT) in pure cultures of *E. coli* [31,32]. Therefore, it is deduced that NPs may promote ARGs spread by affecting mobile genetic elements (MGEs) in the environment, which contradicts the above assumptions that NPs may reduce ARGs by reducing bacterial abundance. Regrettably, current knowledge can hardly confirm whether NPs application is feasible for reducing ARGs dissemination at site remediation operations.

The abuse of antibiotics has caused large amounts of antibiotics to be discharged into the environment, which may induce the amplification of ARGs [33,34]. Furthermore, NPs are reported to be able to inactivate and remove antibiotics from an aqueous solution [35,36]. Thus, it is assumed that NPs may reduce the inductive effects of antibiotics on amplifying ARGs, which may partially reduce ARGs in the environment. However, the effectiveness of the coexistence of antibiotics and NPs on ARGs spread is not clear at present. Therefore, examining the effect of NPs on ARGs in the environment is necessary to understand whether NPs could reduce ARGs dissemination in the environment with or without antibiotics as an inducer.

This study hypothesizes that NPs have dual roles in affecting ARGs spread because of inhibiting bacterial abundance/community via VGT and promoting ARGs dissemination via HGT. In addition, NPs may reduce the effectiveness of antibiotics in inducing ARGs. For testing these hypotheses, this study investigated the effect of NP addition on ARGs/MGEs abundance and bacterial community in sediment overlaid without and with antibiotics solution. Tetracycline resistance genes (tet-ARGs) and the first-class integron (*intI1*) were chosen to serve as representative markers of ARGs and MGEs, respectively. Moreover, this study chose nZVI and nZnO to represent NPs, and tetracycline as a representative antibiotic because of its wide usage and existence in water environments.

## 2. Materials and Methods

### 2.1. Sediment and Sampling

Sediment was sampled from a lake located in Chengdu, Sichuan Province, China. The longitude and latitude of the sampling site are 104°16′42″ E and 30°32′23″ N. This lake is used as a backup drinking water supply and stores approximately 2.8 million m^3^ of water. The water quality achieved Class III of the National Surface Water Quality Standard of China, indicating the water meets the requirement of second-grade drinking water, and for fisheries [37]. Briefly, the sediment was collected using a sediment grab for the surface sediment. After sampling, sediment was mixed thoroughly and passed through 10 mesh sieves (2 mm) to remove large debris, rock, and stones. The contents of total carbon and nitrogen in sediment were measured by an elemental analyzer (Vario El III, Elementar, Hanau, Germany), while the total phosphorus content was determined by melt-molybdenum, antimony, and scandium colorimetry [38]. The total carbon, nitrogen, and phosphorus contents were 0.5, 0.06, and 2.1 mg g^−1^, respectively.

### 2.2. Basic Properties of Nanoparticles

In this study, nZVI and nZnO were chosen as representative NPs. The nominal sizes of nZVI and nZnO were approximately 50 nm and 20 nm, respectively, according to Shanghai Xiangtian Co. Lit. (Shanghai, China). The BET surface area and pore volume of nZVI and nZnO were 26.4 m^2^ g^−1^ and 30.1 m^2^ g^−1^, and 0.015 and 0.076 cm^3^ g^−1^, respectively. The morphology of nZVI differed from nZnO, with nZVI, being a regular sphere, while nZnO had an irregular rod shape (Figure S1).

### 2.3. Incubation Experiments

Microcosm and incubation were used to investigate the addition of nanoparticles on ARGs under two scenarios: (1) sediment only (no tetracycline in the underlying water), and (2) sediment amended with tetracycline (10 mg L^−1^ tetracycline in the overlying water). The addition of nanoparticles into the sediment was set at 0, 0.0035, 0.035, 0.35, 3.5, and 35 mmol kg^−1^. The abbreviations of the different treatments are presented in Table 1. Briefly, 100 g sediment or sediment added with NPs (dry weight basis), was put into a sterilized 500 mL serum bottle (Figure S2). Then, 250 mL of overlying water was added to the bottle. In Scenario 1, overlying water was prepared with distilled water, while overlying water containing 10 mg L^−1^ tetracycline was applied in Scenario 2. The incubation was set at 25 °C for 28 days in the dark. Each treatment was conducted in triplicate. After incubation, the concentration of tetracycline in the overlying water was determined, but not detected in all treatments. The nZVI and nZnO were almost undissolved with soluble proportions (e.g., Fe^2+^ or Zn^2+^) in the biological media lower than 0.003% during incubation. Detailed dissolution information is listed in Table S1.

### 2.4. Quantitative-PCR and High-Throughput Sequencing

After incubation, the sediment in each serum bottle was collected for DNA extraction. According to the instruction manual, total DNA was extracted by DNeasy PowerSoil Kit (QIAGEN, Leipzig, Germany). The extracted DNA was checked for quality using Nanodrop 2000 (Thermo, Waltham, MA, USA) to ensure the value of A_260–280_ ranged from 1.8 to 2.0, and the DNA concentration was higher than 10 ng μL^−1^. Afterward, the DNA was stored at −80 °C before further analysis [17].

This study chose tet-ARGs and *intI1* as representative marker genes to explore the NP additions on the fate of ARGs. Target genes, including *tetA*, *tetC*, *tetM*, *tetO*, *tetQ*, *tetW*, *tetX*, *intI1* and 16S rRNA, were quantified by quantitative-PCR. The primers used for detecting these genes and the procedure conditions are listed in Table S2. A PCR Thermal Cycler Dice Real-Time System (Applied Biosystems, Foster City, CA, USA) was applied to conduct a quantitative-PCR procedure. The PCR procedure was as follows: 2 min initial denaturation at 95 °C followed by 40 cycles of denaturation at 95 °C for 10 s, and anneal and extended at 60 °C for 40 s [17]. The absolute abundance of target genes was represented as Log_10_ (gene copies) g^−1^ dry sediment.

Extracted genomic DNA of high quality was sent to Chengdu Institute of Biology, Chinese Academy of Sciences, for high-throughput sequencing (Illumina Miseq, San Diego, CA, USA). FLASH software (version 1.2.7) was applied to remove sequences shorter than 20 bp and quality scores lower than 75%. USEARCH software (version 8.0) was applied to cluster sequences. Finally, the operational taxonomic units (OTUs) with 97% similarity were assigned on the QIIME platform via the RDP Classifier [39].

### 2.5. Data Analysis

The data were analyzed by Excel software (version 2016, Microsoft, Redmond, DC, USA) and Origin software (version 2018, OriginLab, Northampton, MA, USA). In general, significance was accepted at *p* < 0.05. Moreover, the Partial Least Squares Path Model (PLS-PM) was used to assess the direct and indirect effect of nanoparticles, bacterial abundance, bacterial communities, and horizontal transfer (employing *intI1*) on tet-ARGs spread in sediment. PLS-PM was constructed by the R package plspm (version 0.4.9, R Studio, Boston, MA, USA) [40]. Finally, network analysis was applied to reveal the potential bacterial hosts of tet-ARGs and *intI1* through R, utilizing the “psych” package and visualized via Gephi (version 0.9.2) (https:/gephi.org, accessed on 12 October 2021) [40].

## 3. Results

### 3.1. The Effect of NPs on Tet-ARGs/intI1 Abundance in Sediments

**Figure 1:**
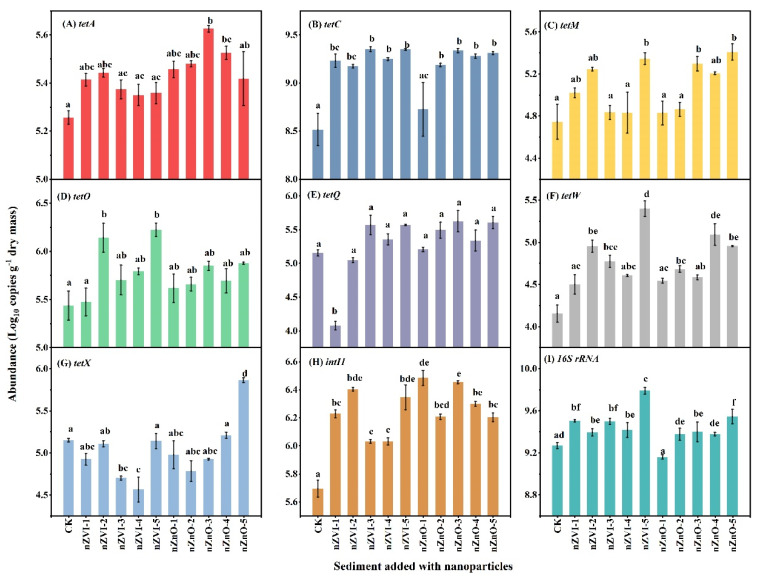
Effect of nanoparticles on the abundance of (**A**) *tetA*, (**B**) *tetC*, (**C**) *tetM*, (**D**) *tetO*, (**E**) *tetQ*, (**F**) *tetW*, (**G**) *tet*X, (**H**) *intI1* and (**I**) 16S rRNA in sediment under microcosm incubation (scenario 1). The different letter indicates *p* < 0.05.

The results related NP addition on tet-ARGs/*intI1* and 16S rRNA abundance in sediment incubation microcosms (i.e., scenario 1) are presented in Figure 1. Both the type and concentration of NPs influenced the selected genes’ abundance (Figure 1A–I). The abundance of *tetA*, *tetC*, *tetM*, *tetO*, *tetQ*, *tetW*, *tetX*, *intI1* and 16S rRNA in CK were 5.26, 8.52, 4.75, 5.44, 5.16, 4.15, 5.15, 5.70, and 9.27 logs, respectively (Figure 1A–I). Compared to CK, nZVI additions increased the abundance of *tetA*, *tetC*, *tetM*, *tetO*, *tetW*, *intI1* and 16S rRNA by 0.09-0.18, 0.65-0.83, 0.08-0.60, 0.03-0.78, 0.35-1.25, 0.33-0.65, and 0.13-0.52 logs, respectively (Figure 1A–D,F,H,I). On the other hand, nZnO additions increased the abundance of *tetA*, *tetC*, *tetM*, *tetO*, *tetQ*, *tetW*, and *intI1* by 0.16-0.37, 0.21-0.82, 0.08-0.66, 0.18-0.44, 0.05-0.47, 0.40-0.94,and 0.50-0.78 logs relative to CK, respectively (Figure 1A–F and H).

When the abundance of all tet-ARGs was calculated as a sum, the total abundance of tet-ARGs after nZVI and nZnO amendment (38.6-42.4 logs) was higher than the control sediment (CK) (38.4 logs) (Figure 1A–I). The highest increment of tet-ARGs was found in nZVI-5 (3.97 logs) and nZnO-5 (4.02 logs) added sediment. Besides, it should be noted that the abundance of *intI1* was significantly increased regardless of the concentrations of the NPs in this study (*p* < 0.05) (Figure 1G). Different from *intI1*, the 16S rRNA gene was only increased significantly by nZVI addition and higher addition of nZnO (e.g., nZnO-5) (*p* < 0.05) (Figure 1I). By comparison, the increment of *intI1* abundance (0.34 to 0.79 logs) was higher than 16S rRNA (−0.11 to 0.52 logs). Based on these findings, the nZVI and nZnO additions affected these genes differently and generally increased favorably for tet-ARGs, *intI1*, and bacterial abundance.

### 3.2. The Effect of NPs on the Inducing Role of TC in Tet-ARGs/intI1 Abundance in Sediment

**Figure 2:**
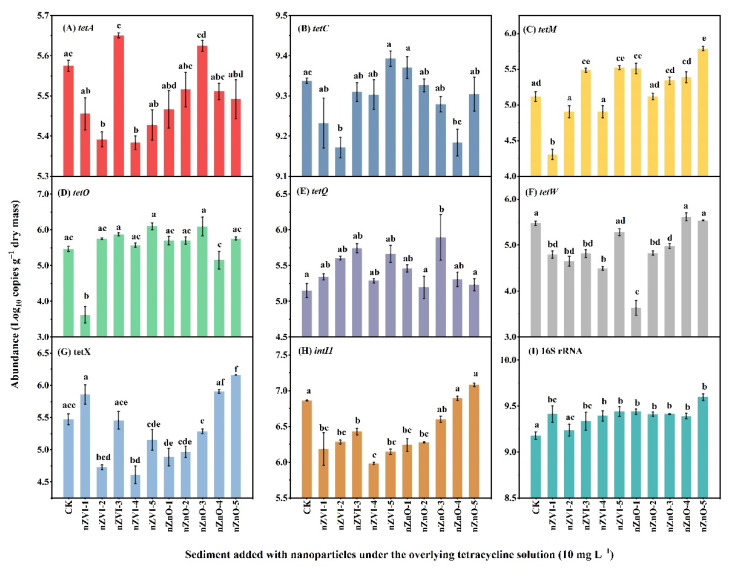
Effect of nanoparticles on the abundance of (**A**) *tetA*, (**B**) *tetC*, (**C**) *tetM*, (**D**) *tetO*, (**E**) *tetQ*, (**F**) *tetW*, (**G**) *tet*X, (**H**) *intI1* and (**I**) 16S rRNA sediment overlaid with 10 mg L^−1^ tetracycline solution (scenario 2). Different letters indicate *p* < 0.05.

The effect of NPs on the inducing role of TC in tet-ARGs/*intI1* abundance in sediment (i.e., scenario 2) is shown in Figure 2. In CK+TC sediment, the abundance of *tetA*, *tetC*, *tetM*, *tetO*, *tetQ*, *tetW*, *tetX*, *intI1* and 16S rRNA in CK were 5.57, 9.34, 5.12, 5.46, 5.15, 5.48, 5.47, 6.86, and 9.18 logs, respectively (Figure 2A–I). Compared to CK+TC, nZVI additions reduced the abundance of *tetW*, *tetX* and *intI1* by 0.20-0.98, 0.01-0.86, and 0.43-0.88 logs, respectively (Figure 2F–H). Notably, lower nZnO additions (nZnO-1, nZnO-2, nZnO-3) reduced the abundance of *tetW*, *tetX*, and *intI1* by 0.50-1.85, 0.18-0.58, and 0.26-0.62 logs, while higher nZnO additions (nZnO-4, nZnO-5) promoted the abundance of these genes (Figure 2F–H). It should be noted that nZVI and nZnO additions did not reduce, but increased, the abundance of 16S rRNA by 0.06-0.42 logs (Figure 2I).

The sum of tet-ARGs in CK+TC was 41.59 logs, while the sum of tet-ARGs abundance in NPs sediments with TC ranged from 38.61 to 43.27 logs (Figure 2A–G). Notably, the highest reduction by nZVI and nZnO additions was found in nZVI-1 (2.98 logs) and nZnO-1 (1.57 logs), respectively. On the other hand, the highest addition of nZVI (nZVI-5) and nZnO (nZnO-5) increased the sum of tet-ARGs by 0.94 logs and 1.68 logs, respectively. Collectively, it can be concluded that nZVI and nZnO could reduce and increase the abundance of tet-ARGs depending on NPs dosage and type.

### 3.3. The Effect of nZVI and nZnO on Bacterial Diversity and Communities

The impact of nZVI and nZnO on bacterial diversity is presented in Table 2. In Scenario 1, nZVI and nZnO additions reduced OTUs, Shannon, and Chao1 index (Table 2). Relative to CK, nZVI-5 and nZnO-5 addition reduced OTUs, Shannon, and Chao1 index by 27.2%, 13.2%, 29.4%, and 39.3%, 19.0%, and 37.2%, respectively. In Scenario 2, nZVI and nZnO additions generally reduced bacterial diversity, except for nZVI-1and nZnO-1 (Table 2). Compared to CK+TC, nZVI-5 and nZnO-5 addition reduced OTUs, Shannon, and Chao1 index by 27.1%, 14.5%, 29.1%, and 33.7%, 18.2%, 36.4%, respectively.

**Figure 3:**
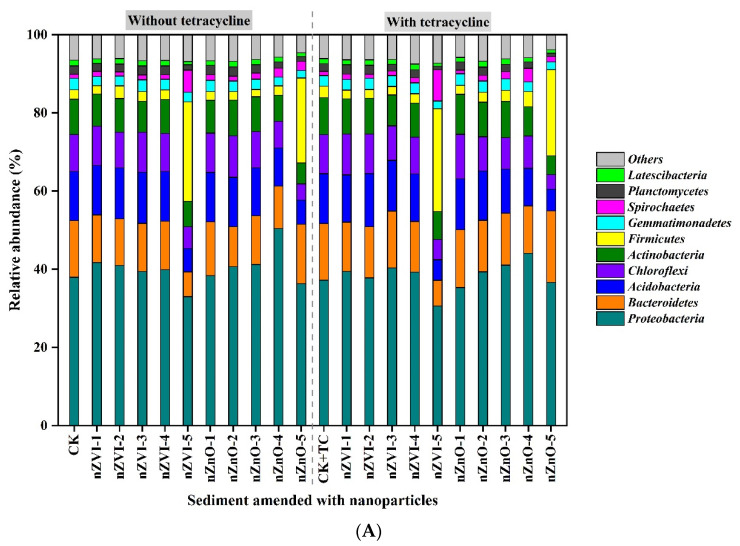

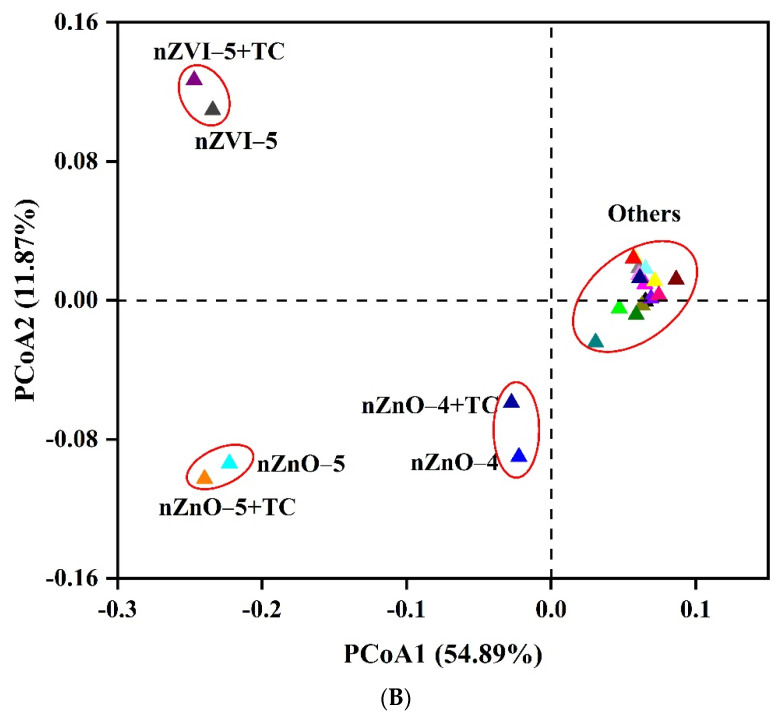
(**A**) Bacterial community composition at phylum level in nanoparticle-amended sediments. (**B**) PCoA analysis of bacterial communities under nanoparticles addition and coexistence of nanoparticles and tetracycline in sediment. Others include CK, CK, nZnO-1, nZnO-2, nZnO-3, nZVI-1, nZVI-2, nZVI-3, nZVI-4, and their counterparts in scenario 2 (+TC).

The bacterial communities at the phylum level and their differences are presented in Figure 3. The relative abundance of *Firmicutes* in sediment was increased after nZVI-5 and nZnO-5 addition, while the relative abundance of *Acidobacteria* was decreased (Figure 3A). The reduction of *Bacteroidetes* was observed in nZVI-5 sediment but not in nZnO-5 sediment (Figure 3A). The PCoA results (Figure 3B) showed that bacterial communities in nZVI-5, nZnO-4, and nZnO-5 differed greatly from each other and other sediments, while the other sediments were close to each other. These findings suggested that adding a higher concentration of NPs (i.e., nZVI-5, nZnO-4, and nZnO-5) altered the bacterial community structure in sediment. Notably, it was found that sediments without TC were close to their counterpart with TC (Figure 3B). For example, nZVI-5 was close to nZVI-5+TC, nZnO-4 was close to nZnO-4+TC, and nZnO-5 was close to nZnO-5+TC (Figure 3B), suggesting that TC did not change the bacterial community structure remarkably.

### 3.4. The Correlations Related Tet-ARGs to NPs, intI1 Abundance, Bacterial Abundance and Community

**Figure 4:**
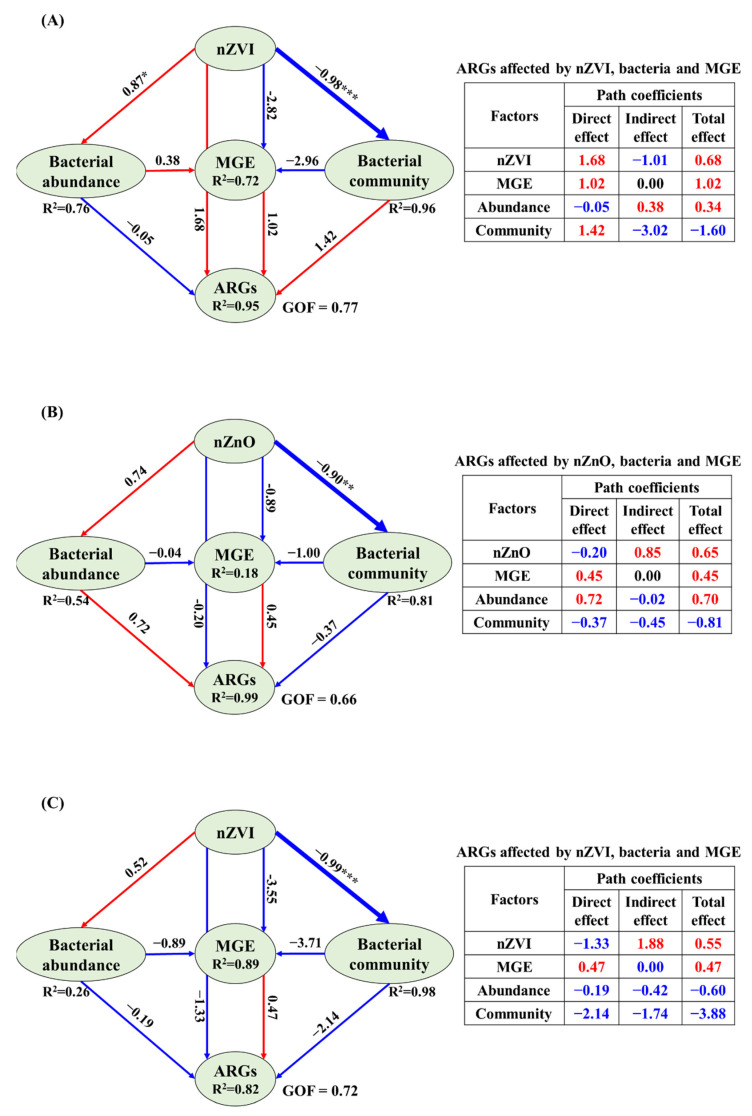

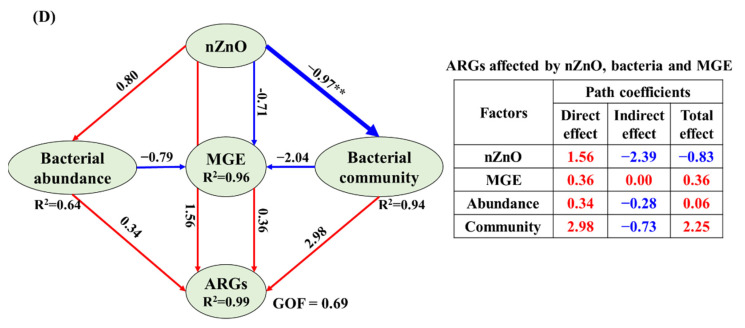
The partial least squares path model (PLS-PM) shows different factors′ direct and indirect effects on ARGs abundance in sediment. (**A**) sediment added with nZVI but without tetracycline, (**B**) sediment added with nZnO but without tetracycline, (**C**) sediment added with nZVI and overlaid with 10 mg L ^−1^ tetracycline, and (**D**) sediment added with nZnO and overlaid with 10 mg L ^−1^ tetracycline. Red indicates a positive effect, while blue represents a negative effect. *, **, and *** represent *p* < 0.05, *p* < 0.01 and *p* < 0.001, respectively. Abundance indicates bacterial abundance, and community represents bacterial community.

The correlations related tet-ARGs to NPs, *intI1* abundance, bacterial abundance, and community were explored by PLS analysis to find out the possible reasons why and how NPs affected tet-ARGs spread (Figure 4). In general, nZVI and nZnO could directly affect tet-ARGs (i.e., repression between NPs and ARGs); and indirectly affect tet-ARGs via affecting MGE (*intI1*), bacterial abundance, and bacterial community (i.e., repressions of multiple variables) (Figure 4).

In Scenario1 (without TC), the most direct effect on tet-ARGs in nZVI sediment was nZVI (1.68) (Figure 4A). The direct effect of the bacterial community (1.42) and MGE (1.02) on tet-ARGs was also high. Compared to MGE, bacterial abundance slightly affected tet-ARGs (−0.05) (Figure 4A). Unlike nZVI sediment, the most direct effect on tet-ARGs in nZnO sediment was bacterial abundance (0.72), while the direct effect of nZnO on tet-ARGs was small (−0.27) (Figure 4B). Except for bacterial abundance, MGE directly affected tet-ARGs with a value of 0.45 (Figure 4B). Notably, although the direct or indirect effects of these factors on tet-ARGs were given, no significance (*p* > 0.05) was observed.

In Scenario 2 (with TC), both nZVI (−1.33) and bacterial community (−2.14) showed a higher and negative effect on tet-ARGs in nZVI sediment, even though insignificant (*p* > 0.05) (Figure 4C). Unlike nZVI, nZnO (1.56) and bacterial community (2.98) showed a higher and positive effect (*p* > 0.05) on tet-ARGs in nZnO sediment under Scenario 2 (Figure 4D).

### 3.5. The Network Analysis between tet-ARGs/intI1 and the Bacterial Community at the Genus Level

**Figure 5:**
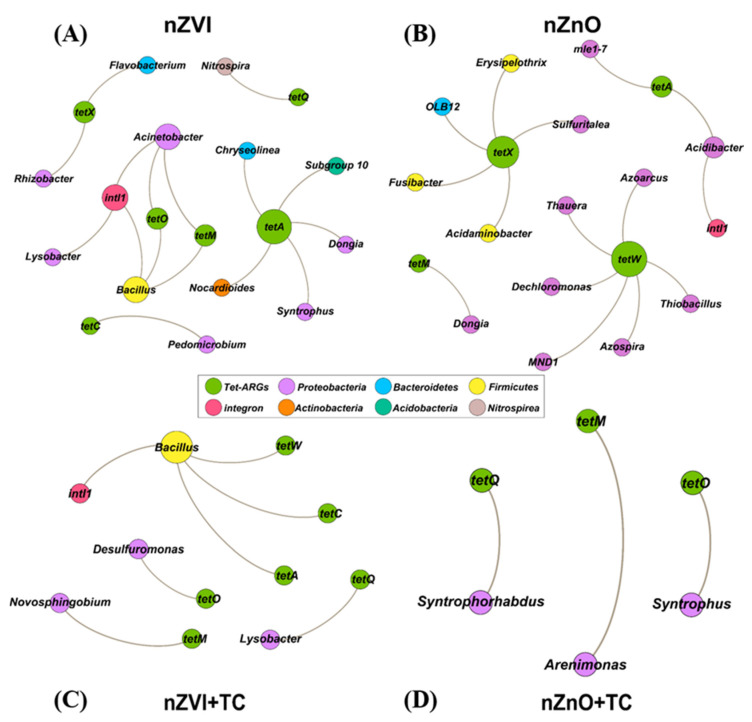
Network analysis between bacterial genera and *tet*-ARG/*intI1* in (**A**) nZVI, (**B**) nZnO, (**C**) nZVI+TC, and (**D**) nZnO+TC sediment. TC means sediment overlaid with 10 mg L^−1^ tetracycline solution.

Network analysis between tet-ARGs/*intI1* and the bacterial community at the genus level (Top 50) was further explored to reveal the potential hosts of these genes under different treatments (Figure 5). There were 50 genera used to analyze the possible hosts of ARGs; thus, Figure 5 only presents the correlations between tet-ARGs/intI1 and bacterial genera with Pearson r > 0.6 with significance at *p* < 0.05. In Scenario 1 (without TC), 12 genera were significantly correlated with seven tet-ARGs/*intI1* in nZVI sediments (Figure 5A), and 13 genera were strongly related to five tet-ARGs/*intI1* in nZnO sediments (Figure 5B). In Scenario 2 (TC), four bacterial genera were significantly correlated with seven tet-ARGs/*intI1* in nZVI+TC sediment (Figure 5C), while three genera were significantly related to three tet-ARG in nZnO+TC sediments (Figure 5D). *Proteobacteria*, *Bacteroidetes*, and *Firmicutes* were the potential hosts of tet-ARGs/*intI1* at the phylum level (Figure 5).

## 4. Discussion

### 4.1. NPs Promote Tet-ARGs/intI1 Abundance in Sediment

According to previous studies, nZVI and nZnO addition may be toxic to bacterial growth, causing a reduction in bacterial abundance [41,42,43,44,45]. If bacterial biomass/growth is reduced after NPs addition, tet-ARGs replication via VGT should be decreased accordingly. However, this study observed that the abundance of bacteria and tet-ARGs were increased by nZVI and nZnO additions (Figure 1), contradicting the hypothesis that NPs should reduce bacterial abundance. In fact, no impact of NPs on microbial abundance was also reported when NP addition to sediment was executed at a lower dosage (e.g., 100 mg g^−1^) [46]. In this study, most nZVI and nZnO additions to sediment were much lower than those reported in studies that ranged from 2000 to 34,000 mg kg^−1^ soil [42,47]. Therefore, the lower dosage of nZVI and nZnO did not reduce but increased, bacterial abundance. In general, nZVI and nZnO can adsorb or remove pollutants that inhibit bacterial growth [24], thus might indirectly promoting bacterial growth.

This study also observed that nZVI and nZnO addition significantly increased the abundance of *intI1* (essential HGT indicators) (Figure 1H), which agrees with the hypothesis that NPs promote the HGT of ARGs. In recent years, NPs (e.g., copper, silver, zinc oxide, titanium dioxide, and alumina NPs) were found to facilitate conjugative transfer (one kind of HGT) of plasmid-mediated ARGs across bacterial genera, suggesting the risk of NPs for disseminating of ARGs [31,32,48,49]. Moreover, Zhang et al. [50] proposed that the promoting effect of NPs on HGT of ARGs is comparable to the inducing effect of antibiotics, further emphasizing the critical role of NPs in spreading ARGs in the environment. It should be noted that most of these studies were conducted based on pure cultures of bacteria; however, whether NPs could promote the HGT of environmental ARGs, which has significant implications for understanding ARG fate in the environment, is still unknown. Fortunately, this study revealed that nZVI and nZnO addition promoted the role of HGT in disseminating ARGs. More importantly, it seems that the role of HGT in disseminating ARGs was more vital than VGT since the increased abundance of *intI1* by nZVI and nZnO addition was higher than 16S rRNA (Figure 1H,I).

This study also observed that the influence of the same NPs on different tet-ARGs was different (Figure 1). The primary reason is that tetracycline resistance mechanisms differ for these specific genes. For example, *tetA* and *tetC* are attributed to efflux pumps [51,52]; *tetM*, *tetO*, *tetQ*, and *tetW* are attributed to ribosomal protection proteins [52,53]; and *tetX* is related to enzymatic inactivation [52,54]. Furthermore, these genes are bacteria-specific [52], contributing to the different changes of tet-ARGs by the same NP addition. In addition, the impact of nZVI and nZnO on the same *tet*-ARG was different at the same concentration, which was mainly caused by their various physicochemical properties, such as the core materials, particle size, and morphology (Figure S1) [41,50].

### 4.2. NPs Reduce the Role of TC in Inducing tet-ARGs/intI1 in Sediment

Compared with the counterparts of these genes shown in Figure 1, the abundance of tet-ARGs/*intI1* in Scenario 2 (i.e., with TC) was higher than in Scenario 1 (i.e., without TC) (Figure 1 and Figure 2), implying TC induced the propagation of tet-ARGs/*intI1*. This is not a surprise because previous studies have reported that the existence of TC could enhance tet-ARGs abundance [55,56,57,58]. In addition, it is well known that bacteria can adopt tet-ARGs via efflux pump (*tetA* and *tetC*) or a mechanism involving ribosomal protection proteins (*tetM*, *tetO*, *tetQ,* and *tetW*), under the pressure of TC [52,55,56]. Thus, it is reasonable for TC to induce a higher abundance of these tet-ARGs (Figure S3).

Currently, the coexistence of antibiotics and NPs related to the fate of ARGs in the environment is an emerging issue that has been less reported. However, there is information that NPs can inactivate and remove antibiotics from aqueous solutions by different ways, such as adsorption, oxidation, and reduction [22,59,60]. For example, nZVI removes 90% TC under neutral conditions via adsorption [60], while nZnO modified adsorbent exhibits a high adsorption capacity for TC (approximately 98.7 mg g^−1^) [22]. TC in nZVI and nZnO sediments was not detected in this study (data not shown). Collectively, nZVI and nZnO addition can adsorb TC and then reduce TC mobility, thus alleviating the pressure of TC on bacteria and reducing the role of TC in inducing tet-ARGs. Notably, higher NPs addition should be avoided because the role of NPs in promoting tet-ARGs/*intI1* might exceed the inducing role of TC; for instance, in scenario 2, nZnO-5 addition significantly promoted *tetM* and *tetX* relative to CK+TC (Figure 2).

### 4.3. NPs Alter Bacterial Diversity and Communities in Sediment

Currently, the impact of NPs on microorganisms in sediment is rarely reported, whereas this work partially supplemented the missing information. It is well known that environmental microorganisms are often resilient to perturbations (e.g., metal oxide NPs) [41]. Therefore, if considering the NPs as perturbations, the lower perturbation might not significantly result in microorganism changes; however, microorganisms may change when the perturbation becomes stronger [41]. Consequently, it is plausible that lower nZVI and nZnO addition did not change the bacterial community, while higher nZVI and nZnO additions strongly altered the bacterial community (Figure 3).

NPs could be either beneficial or detrimental to the microbial communities depending on the kind and concentration of NPs [24,27,44,45]. Usually, the interactions between NPs and bacteria relate to the disruption of membrane integrity and semipermeability, DNA or protein damage, bacterial agglomeration, and toxicity [24,44,45]. Given the strong reducing power of nZVI, the primary mechanism of nZVI influencing bacteria may be oxidative stress that destroys membrane properties and causes DNA damage [21,25,61]. In contrast, nZnO entering the environment is frequently found to generate reactive oxygen species (ROS) [42,62,63]; thus, the destruction of membrane semipermeability and DNA or protein damage by ROS is proposed as the main reason for bacterial alteration in sediment.

Notably, the effect of NPs on microorganisms is species-specific. As shown in Figure 3A, nZVI reduced the relative abundance of *Acidobacteria* and *Bacteroidetes,* indicating that these bacteria were more easily destroyed by nZVI. Additionally, the relative abundance of *Firmicutes* was increased by both nZnO and nZVI addition, suggesting that these bacteria were more resistant to nZnO and nZVI disturbance. These results implied that *Firmicutes* may play an essential role in spreading tet-ARGs/*intI1*, consistent with our previous studies [16,17]. By comparison, nZnO seemed more toxic to bacteria than nZVI because a lower concentration of nZnO (e.g., nZnO-4) also significantly altered the bacterial community (Figure 3B). The possible reason is that nZnO has a smaller particle size, making nZnO enter the bacterial cells more easily and obstructing the reproduction of certain bacteria [63].

The bacterial community under Scenario 2 (with TC) was similar to its counterpart in Scenario 1 (without TC) (Figure 3). However, unlike this work, published research reported that TC inhibited soil microbial growth and changed soil microbial community and function by selecting TC-resistant bacteria and decreasing sensitive bacterial groups [64,65]. Since TC was possibly adsorbed by sediment organic matter or NPs, the inhibitory role of TC was correspondingly reduced, causing insignificant changes in bacterial community structure in the present study.

### 4.4. Potential Reasons for ARGs Spread Affected by NPs in Sediment

As shown in Figure 4A, in scenario 1, the relative effect of factors on ARGs in nZVI sediment was nZVI (1.68) > bacterial community (1.42) > MGE (1.02) > bacterial abundance (−0.05). nZVI may result in oxidative stress on bacteria due to its strong reducing power, thus causing bacteria to become resilient and produce resistance genes [21,25,41,61]. The lower effect of bacterial abundance compared to MGE indicates that HGT among different bacteria might be more critical than VGT in nZVI sediment. The reasons why nZVI promoted HGT of ARGs can be attributed to: (1) the over-production of ROS caused by nZVI addition; (2) the up-regulated expressional levels of genes and proteins related to oxidative stress, cell membrane damage, and pilus generation, and (3) carrying ARGs for transmembrane transport (carries) [10,31,66]. Nonetheless, in this study, it was not a simple task to identify the reason for inducing HGT to increase tet-ARGs since sediment organic matter can affect the fate of tet-ARGs and nZVI [41].

In scenario 1, the relative effect of factors on ARGs in nZnO sediment were bacterial abundance (0.72) > MGE (0.45) > bacterial community (−0.37) > nZnO (−0.27) (Figure 4B). Bacteria are carriers of ARGs [44,45]; therefore, bacterial abundance positively affected tet-ARGs via VGT. Except for VGT, HGT also contributed partially to the spread of tet-ARGs in nZnO sediment. Previous studies reported that nZnO could promote spread across pure bacteria via HGT [31]. Under realistic conditions, the fate of environmental ARGs possibly differed from that of pure bacteria since the interactions between sediment and NPs might strongly affect the role of NPs in spreading ARGs. Coincidently, this study provided information that nZnO promoted the spread of ARGs across bacteria via HGT in sediment, which is vital for predicting the risk of ARGs in the environment.

In scenario 2, the negative effect of nZVI (−1.33) on ARGs might be because nZVI reduced the role of TC in inducing tet-ARG by adsorbing TC (Figure 1, Figure 2 and Figure 4C) [60]. On the other hand, the negative effect of the bacterial community (−2.14) (Figure 4C) on ARGs might be because the hosts of tet-ARGs were possibly reduced by nZVI, thus causing a reduction of tet-ARGs. The reduction of certain bacteria (e.g., *Acidobacteria* and *Bacteroidetes*) in Figure 3 partially supported the reason.

In scenario 2, nZnO (1.56) and bacterial community affected ARGs more than bacterial abundance and MGE in nZnO sediment (Figure 4D). The increased *Firmicutes* (e.g., nZnO-4+TC and nZnO-5+TC) in Figure 3 may explain the positive effect of the bacterial community on ARGs in nZnO sediment. Compared to nZVI, nZnO has a higher surface area and pore volume but a smaller size (Figure S1). Generally, the larger the surface area and pore volume, the higher the pollutants’ adsorption is [67]. Therefore, nZnO might still exert stress on bacteria and promote the production of resistance, although nZnO could inhibit the role of TC in inducing tet-ARGs.

### 4.5. The Potential Hosts of Tet-ARGs in Different NPs Added Sediments

In scenario 1, comparing nZVI and nZnO sediment (Figure 5A,B), bacterial genera carrying tet-ARGs/*intI1* differed widely, suggesting that nZVI and nZnO affected bacteria by different mechanisms, such as the disruption of membrane integrity, DNA or protein damage [21,25,62,63]. However, TC existence reduced and altered the correlations between bacterial community and tet-ARGs/*intI1*. The lost relationships correlated *tetX* with *Proteobacteria* and *Bacetroidetes* revealed that the resistance mechanism of enzymatic inactivation might be lost under the pressure of TC when comparing nZVI and nZVI+TC sediment (Figure 5A,C) [52,68]. Furthermore, only *tetO*, *tetM*, and *tetQ* were significantly correlated with *Proteobacteria* in nZnO+TC sediment (Figure 5D), implying the dominant resistance was ribosomal protection proteins, and other resistance mechanisms might be lost due to the pressure of TC [52,53]. Notably, there was no correlation between *intI1* and bacterial genera in nZnO+TC sediment, implying that TC might destroy the hosts of *intI1* (e.g., *Acidibacter*, which was reduced by the coexistence of nZnO and TC in Figure S4) (Figure 5B,D).

## 5. Conclusions

This study revealed that nZVI and nZnO addition can promote the spread of tet-ARGs/*intI1* in sediment via both VGT and HGT. However, the lower addition of nZVI and nZnO (e.g., <0.035 mmol kg^−1^) can reduce tet-ARGs/*intI1* dissemination in sediment if the overlying water contains tetracycline. Moreover, a higher addition of nZVI and nZnO strongly altered bacterial community compositions regardless of whether tetracycline was present. By comparison, the effect of nZnO on ARGs and bacteria differed from nZVI, and nZnO may alter the bacterial community and promote ARGs more effectively. Based on these findings, the types and dosage of NPs and environmental background (e.g., whether antibiotics existed or not) should be carefully considered when applying NPs for pollution remediation.

## Figures and Tables

**Table 1:** Experimental design of NP addition and the abbreviation used in this study.

NPs	Concentration (mmol kg^−1^)	Scenario 1: Sediment Only	Scenario 2: Sediment Overlaid with Tetracycline Solution (10 mg L^−1^)
	0	CK	CK+TC
nZVI	0.0035	nZVI-1	nZVI-1+TC
	0.035	nZVI-2	nZVI-2+TC
	0.35	nZVI-3	nZVI-3+TC
	3.5	nZVI-4	nZVI-4+TC
	35	nZVI-5	nZVI-5+TC
nZnO	0.0035	nZnO-1	nZnO-1+TC
	0.035	nZnO-2	nZnO-2+TC
	0.35	nZnO-3	nZnO-3+TC
	3.5	nZnO-4	nZnO-4+TC
	35	nZnO-5	nZnO-5+TC

TC = tetracycline; CK = sediment without nanoparticles and tetracycline; TC+CK indicates sediment with tetracycline but without nanoparticles.

**Table 2:** Changes in bacterial OTUs, diversity, and richness index by nanoparticle addition in sediment with or without tetracycline.

	Scenario 1: Sediment Only			Scenario 2: Sediment with Tetracycline (+TC)		
	OTUs	Shannon	Chao1	OTUs	Shannon	Chao1
CK	10,364	12.59	17,814	10,019	12.51	17,181
nZVI-1	8230	11.90	12,665	10,304	12.59	17,840
nZVI-2	7913	11.85	12,280	9820	12.49	15,962
nZVI-3	9473	12.31	15,975	9496	12.40	15,428
nZVI-4	9790	12.49	15,969	10,418	12.61	17,982
nZVI-5	7547	10.93	12,584	7306	10.70	12,177
nZnO-1	9691	12.47	15,823	10,122	12.56	16,829
nZnO-2	10,247	12.64	15,614	8630	12.17	12,814
nZnO-3	10,260	12.56	17,784	9936	12.47	17,027
nZnO-4	8164	11.89	12,500	9190	12.20	15,385
nZnO-5	7127	10.33	13,105	6542	10.22	10,689

OTUs Indicate Operational Taxonomic Units

## Supplementary Materials

The following supporting information can be downloaded at: https://www.mdpi.com/article/10.3390/ijerph19159405/s1. Figure S1. SEM of nZVI (A 2 μm and B 500 nm) and nZnO (C 2 μm and D 500 nm) used in this study. Figure S2. Diagrammatic sketch of incubation equipment and the incubation condition used in this study. Figure S3. Differences between situation 1 (without tetracycline) and situation 2 (with tetracycline). Figure S4. Relative abundance of bacterial genera under nanoparticles addition and coexistence of nanoparticles and tetracycline in sediment (top 50). Table S1. Soluble proportion of Zn and Fe from nanoparticle (NP-modified sediment after incubation. Table S2. Primer, annealing temperature, and amplification size of the target genes in this study. References [69,70] are cited in the supplementary materials.
