# Supplementary material for: The Dual Roles of Nano Zero-Valent Iron and Zinc Oxide in Antibiotics Resistance Genes (ARGs) Spread in Sediment
Source: Int J Environ Res Public Health. 2022 Jul 31;19(15):9405. doi: 10.3390/ijerph19159405 (PMC9368363; doi:10.3390/ijerph19159405)
Supplement: Supplementary file 1 [file ijerph-19-09405-s001.zip › ijerph-1790706-supplementary.pdf]

International Journal of
Environmental Research and Public Health

The Dual Roles of Nano Zero-Valent Iron and Zinc Oxide in Antibiotics Resistance Genes (ARGs) SPREAD in Sediment

Ling Luo [1,†,*], Dahang Deng [1,†], Xin Zhao [1], Hairong Hu [1], Xinyi Li [1], Ji-Dong Gu [2], Yan He [1], Gang Yang [1], Ouping Deng [3] and Yinlong Xiao [1,*]

[1] College of Environmental Sciences, Sichuan Agricultural University, Chengdu 611130, China; dengdahang@stu.sicau.edu.cn (D.D.); zhaoxin1@stu.sicau.edu.cn (X.Z.); huhairong@stu.sicau.edu.cn (H.H.); li_xinyi@stu.sicau.edu.cn (X.L.); heyan@sicau.edu.cn (Y.H.); yg8813@sicau.edu.cn (G.Y.)
[2] Environmental Science and Engineering, Guangdong Technion-Israel Institute of Technology,
241 Daxue Road, Shantou 515063, China; jidong.gu@gtiit.edu.cn
[3] College of Resources, Sichuan Agricultural University, 211 Huimin Road, Chengdu 611130, China; ouping@sicau.edu.cn
* Correspondence: luoling@sicau.edu.cn (L.L.); xiaoyinlong@sicau.edu.cn (Y.X.)
† These authors contributed equally to this work.

Contents of this file

Figure S1. The SEM of nZVI (A 2 μm and B 500 nm) and nZnO (C 2 μm and D 500 nm) used in this study

Figure S2. The diagrammatic sketch of incubation equipment and the incubation condition used in this study

Figure S3. The differences of tet-ARGs, intI1 and 16S rRNA between situation 1 (without tetracycline) and situation 2 (with tetracycline)

Figure S4. The relative abundance of bacterial genera under nanoparticles addition and co-existence of nanoparticles and tetracycline in sediment (top 50)

Table S1. The soluble proportion of Zn and Fe from the nanoparticles (NPs) modified sediment after incubation

Table S2. The primer, annealing temperature, and amplification size of the target genes in this study

International Journal of
Environmental Research and Public Health

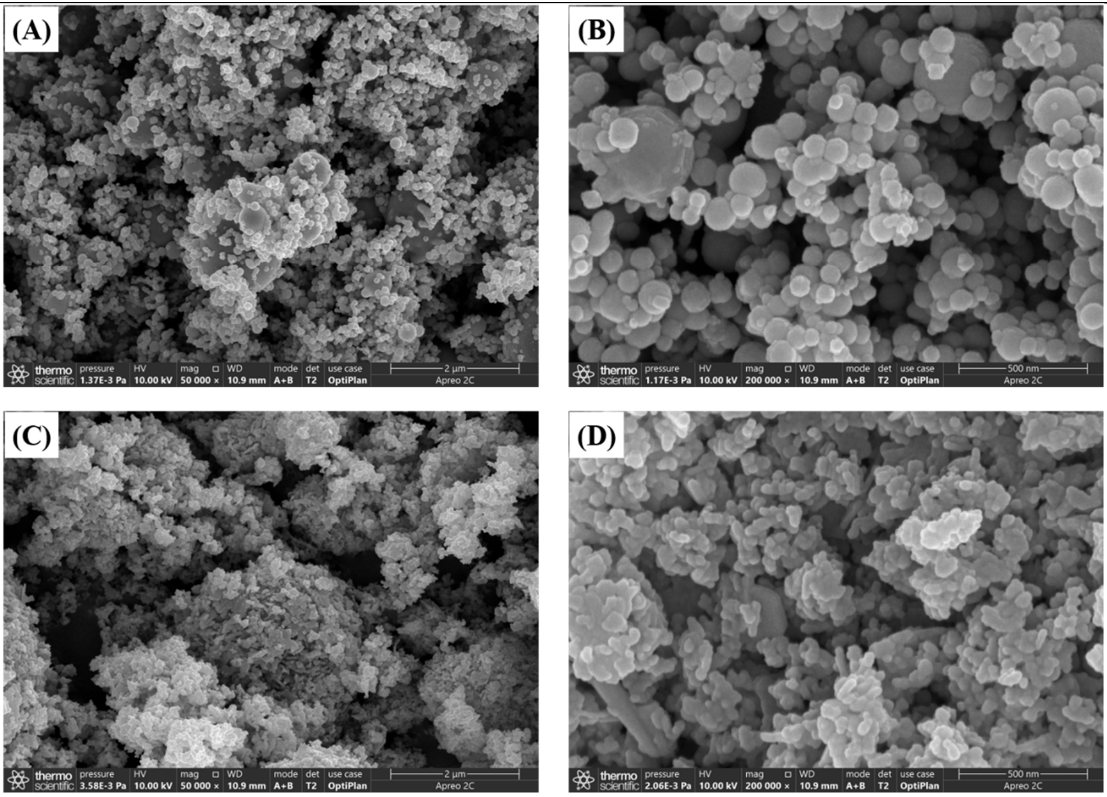

Figure S1. The SEM of nZVI (A 2 μm and B 500 nm) and nZnO (C 2 μm and D 500 nm) used in this study.

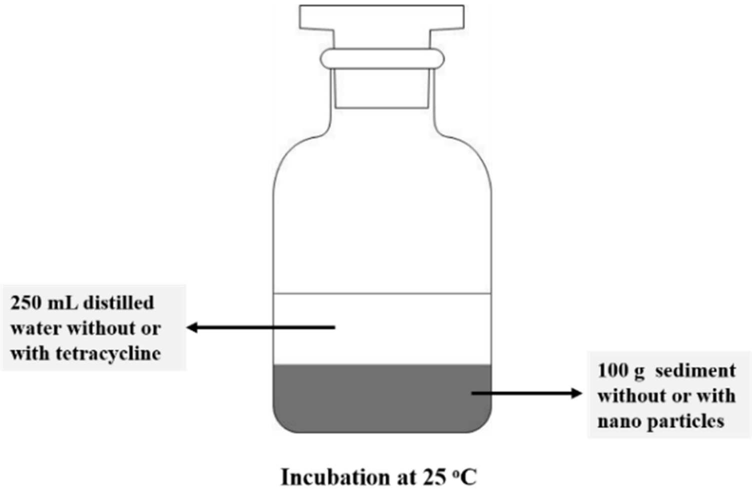

Figure S2. The diagrammatic sketch of incubation equipment and the incubation condition used in this study.

International Journal of
Environmental Research and Public Health

MDPI

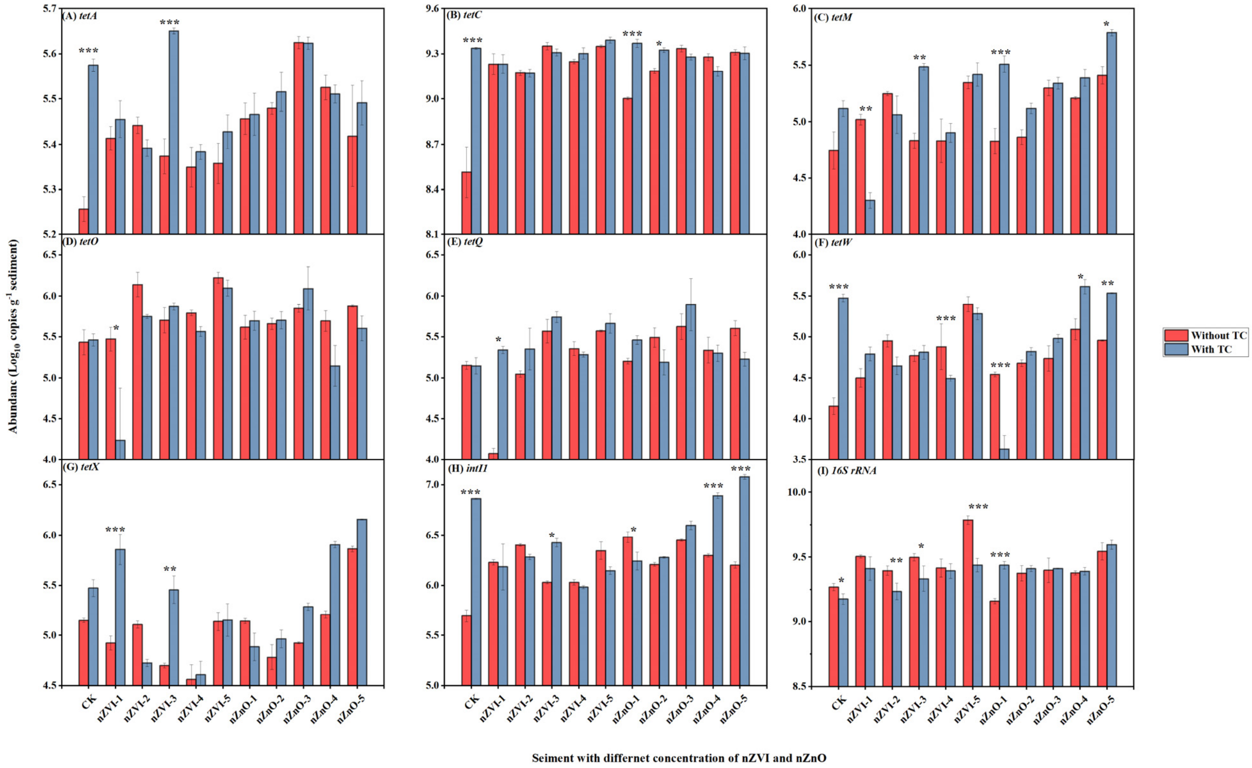

Figure S3. The differences of tet-ARGs, intI1 and 16S rRNA between situation 1 (without tetracycline) and situation 2 (with tetracycline). * $p<0.05$, ** $p<0.01$, and *** $p<0.001$.

International Journal of
Environmental Research and Public Health

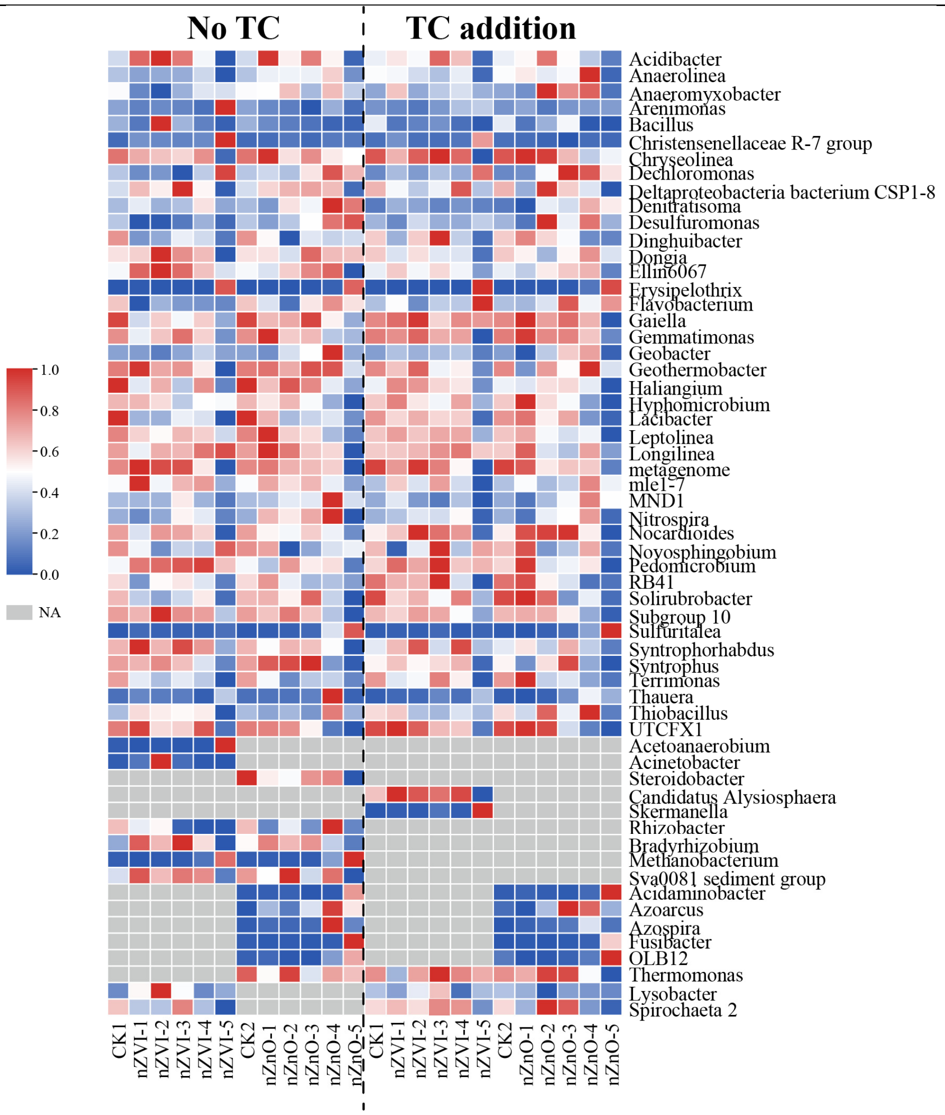

Figure S4. The relative abundance of bacterial genera under nanoparticles addition and co-existence of nanoparticles and tetracycline in sediment (top 50).

International Journal of
Environmental Research and Public Health

Table S1. The soluble proportion of Zn and Fe from the nanoparticles (NPs) modified sediment after incubation.

NPs modified sediment	Dissolved Zn (%)	NPs modified sediment	Dissolved Fe (%)
nZnO-3	0.003	nZVI-3	<0.001
nZnO-5	0.001	nZVI-5	<0.001
nZnO-3+TC	0.003	nZVI-3+TC	<0.001
nZnO-5+TC	0.002	nZVI-5+TC	<0.001

Table S2. The primer, annealing temperature, and amplification size of the target genes in this study.

Target gene	Primer	Sequence (5′-3′)	Amplicon size (bp)	Annealing temperature (°C)	References
tetA	*tetA*-F	GCTACATCCTGCTTGCCTTC	210	60	Chen et al., 2013; Wang et al., 2017 [1,2]
	tetA-R	CATAGATCGCCGTGAAGAGG			
tetC	*tetC-F*	CTTGAGAGCCTTCAACCCAG	418	63	
	tetC-R	ATGGTCGTCATCTACCTGCC			
tetM	*tetM*-F	ACAGAAAGCTTATTATATAAC	171	55	
	tetM-R	TGGCGTGTCTATGATGTTCAC			
tetO	*tetO*-F	GATGGCATA-CAGGCACAGACC	172	57	
	tetO-R	GCCCAACCTTTTGCTTCACTA			
tetQ	*tetQ*-F	AGAATCTGCTGTTTGCCAGTG	169	62	
	tetQ-R	CGGAGTGTCAATGATATTGCA			
tetW	*tetW*-F	GAGAGCCTGCTA-TATGCCAGC	168	60	
	tetW-R	GGGCG-TATCCACAATGTTAAC			
tetX	*tetX-F*	AGCCTTACCAATGGGTG-TAAA	278	60	
	TetX-R	TTCTTACCTTGGACATCCCG			
intI1	*intI1-F*	CCTCCCGCACGATGATC	280	60	
	intI1-R	TCCACGCATCGTCAGGC			
16S rRNA	*515-F*	GTGCCAGCMGCCGCGGTAA	394	56	
	909-R	CCCCGYCAATTCMTTTRAGT			

Reference

1. Chen, B.W.; Liang, X.M.; Huang, X.P.; Zhang, T.; Li, X.D. Differentiating anthropogenic impacts on ARGs in the Pearl River Estuary by using suitable gene indicators. *Water Res.* **2013**, *47*, 2811–2820.
2. Wang, R.; Chen, M.; Feng, F.; Zhang, J.; Sui, Q.; Tong, J.; Wei, Y.; Wei, D. Effects of chlortetracycline and copper on tetracyclines and copper resistance genes and microbial community during swine manure anaerobic digestion. *Bioresour. Technol.* **2017**, *238*, 57–69.
